# Burden of malignant mesothelioma in China during 1990–2019 and the projections through 2029

**DOI:** 10.1016/j.jncc.2024.05.003

**Published:** 2024-05-11

**Authors:** Qiulin Huang, Youli Chen, Liyou Lian, Qiqi Lei, Jinfei Chen, Licun Wu, Kari Hemminki, Jianguang Ji, Tianhui Chen

**Affiliations:** 1 Department of Cancer Prevention, Zhejiang Cancer Hospital, Hangzhou, China; 2 Hangzhou Institute of Medicine (HIM), Chinese Academy of Sciences, Hangzhou, China; 3 School of Public Health, Hangzhou Normal University, Hangzhou, China; 4 State Key Laboratory for Oncogenes and Related Genes; NHC Key Laboratory of Digestive Diseases, Division of Gastroenterology and Hepatology, Shanghai Institute of Digestive Disease, Renji Hospital, School of Medicine, Shanghai Jiao Tong University, Shanghai, China; 5 The First Affiliated Hospital of Wenzhou Medical University, Wenzhou, China; 6 Latner Thoracic Surgery Research Laboratories, Division of Thoracic Surgery, Toronto General Hospital, Princess Margaret Cancer Research Centre, University Health Network, University of Toronto, Toronto, Canada; 7 Biomedical Center, Faculty of Medicine and Biomedical Center in Pilsen, Charles University in Prague, Pilsen, Czech Republic; 8 Division of Cancer Epidemiology, German Cancer Research Center, Heidelberg, Germany; 9 Department of Gynecology Oncology, Fujian Maternity and Child Health Hospital, College of Clinical Medicine for Obstetrics & Gynecology and Pediatrics, Fujian Medical University, Fujian, China; 10 Center for Primary Health Care Research, Lund University, Malmö, Sweden

**Keywords:** Malignant mesothelioma, Cancer burden, China, Global Burden of Disease (GBD) 2019

## Abstract

**Objective:**

To provide the most up-to-date data on the burden of malignant mesothelioma (MM) and the projections through 2029 in China.

**Methods:**

Data on patients diagnosed with MM from China during 1990–2019 were obtained from the Global Burden of Disease (GBD) 2019 database, including annual cases and deaths data and age-standardized rates of incidence, mortality, and disability-adjusted life-years (DALYs) associated with MM among different age groups. Temporal trends during 1990–2019 were analyzed by the Joinpoint regression models using 95% confidence interval (CI), while the projections through 2029 were calculated by the Bayesian age-period-cohort model. Data on the production and consumption of asbestos in China were obtained from the United States Geological Survey on Mineral Commodity Summaries during 1996–2023.

**Results:**

We observed a significant elevation in incident new cases and deaths over the last 3 decades, increasing from 1193 in 1990 to 2815 in 2019 for incident cases and from 1134 in 1990 to 2773 in 2019 for death cases. We found a roughly 6% increase in the proportion of incident cases for those aged >70 years (30% in 2019 versus 24% in 1990), while for the proportion of deaths similar elevation for those aged >70 years was found. Additionally, men had significantly higher DALYs due to MM across age groups compared with women. Asbestos consumption in China dramatically dropped since 2012 and reached the bottom in 2017 with 230 kilotons. By 2029, the projected age-standardized rate for incidence and mortality is expected to reach 1.2 per million for both.

**Conclusion:**

We found, for the first time using GBD data on the Chinese population, that the burden of MM has been significantly increasing in China over the last three decades and will continue to increase in the upcoming decade, suggesting an urgent need for a complete ban on chrysotile asbestos in China.

## 1 Introduction

Malignant mesothelioma (MM) is a rare malignancy originating from serosal membranes, which mainly consists of pleural mesothelioma and peritoneal mesothelioma. The main causes of MM can be attributed to occupational and environmental exposure to asbestos, genetic mutation, and gene-environment interaction.^1^ According to the World Health Organization,^2^ the incidence of mesothelioma has decreased in some developed countries such as Australia, the United States, and certain countries in Western Europe, probably due to the introduction of regulatory laws for asbestos.^3^^,^^4^ However, large amounts of asbestos are still used in many developing countries and much of the previously used asbestos is still in buildings.^5^^,^^6^ Mesothelioma incidence and mortality rates are expected to increase in the coming years due to the ageing of the population, and increased environmental exposure to asbestos from geological sources as rural areas are being developed.^7^

A complete ban on asbestos use is not yet in place in China because chrysotile use is still allowed.^8^ In the past five years, China's asbestos production ranged from 100,000 to 150,000 tons, accounting for approximately 10% of the world's total.^9^ China widely uses chrysotile asbestos, which consists of the majority of asbestos.^10^ A previous study has indicated an increased risk of mesothelioma associated with exposure to chrysotile asbestos, although the relative risk is somewhat lower compared with other types of asbestos.^11^ Specifically, exposure to chrysotile asbestos stands as the leading cause of mesothelioma cases in southeastern China. While the incidence of asbestos-related diseases, especially MM, is expected to increase significantly in China, the peak of MM incidence in China shall be reached much later compared to many industrialized countries owing to the 20–40 years' latency period after asbestos exposure.^12^ In fact, the number of incident cases of MM in China has been reported to increase slowly but consistently over the next few years.^13^ Moreover, limited relevant data are available from the Chinese population and under-reporting is possible due to the poor registration procedure of MM in China.^14^ Therefore, we aimed to examine the burden of MM in China over the past 30 years and to project the burden for 2029 in this study.

## 2 Materials and methods

### 2.1 Data sources

We extracted the MM-related numbers of new cases, deaths, incidence, mortality, and disability-adjusted life-years (DALYs) during 1990–2019 from the Global Burden of Disease (GBD) 2019 online results tool,^15^ which was engineered by the Institute for Health Metrics and Evaluation (IHME). Their respective age-standardized rates (ASRs) were calculated based on the 1966 Segi-Doll world standard population.^16^ The previous studies have reported the general methods of the GBD study in detail.^17^, ^18^, ^19^ The GBD project estimates mortality, years of life lost, years of life lived with disability, and DALYs by age and sex for 87 risk factors and risk factor combinations for 204 countries and territories annually. The 95% uncertainty intervals (UI) of estimated values provided by the GBD study were generated by 1000 draws of the uncertainty distribution with the 2.5th and 97.5th-ordered percentiles.^20^

To assess the burden of MM in China, the researchers accessed various data sources, encompassing censuse data, cancer epidemiology surveys, and information from the Chinese Center for Disease Control and Prevention (CCDC), which included a total of 243 datasets. A comprehensive list for these data sources can be accessed via the Global Health Data Exchange (GHDx) Data Input Sources Tool.^20^ According to the International Code of Diseases 10th (ICD10), MM cases were identified using codes C45-C45.9 in our study.^21^ In the online database, we selected “Deaths,” “DALYs”, and “Incidence” as the measures, “Mesothelioma” as the cause, and “China” as the location for the analyses. In addition, we described the incidence and deaths of MM in China by age and sex and also estimated the percentage changes of these indicators from 1990 to 2019. Besides, the data on the production and consumption of asbestos in China during 1995–2022 were originally obtained from the annual statistics of the United States Geological Survey.^9^

In secondary analyses, we obtained population estimates and projections from the United Nations Department of Economic and Social Affairs Population Division.^22^ The medium-variant projections were used in this study.^23^ The standardized rates of incidence, mortality, and DALYs lost per million person-years of MM were calculated using the age distribution of the Chinese population from the World (WHO 2000–2025) standard.^24^

### 2.2 Assessment of MM burden

The GBD study used a specific methodology to calculate the incidence and mortality of MM,^21^ which could be summarized as below. Firstly, the MM mortality-to-incidence ratio (MIR) was calculated using data on both incidence and mortality. The GBD 2019 database dropped all MIR with less than 10 cases to minimize the impact of statistical noise because MM is a rare cancer with a small sample size, while the threshold for most other cancers was set at 15 cases. Secondly, the incidence of MM was collected from aforementioned data sources. Thirdly, the mortality estimate was derived by multiplying the incidence data by the previously estimated MIR. Finally, the Cause of Death Ensemble model was employed to determine the cancer-specific mortality of MM.^25^^,^^26^

The GBD study used the Comparative Risk Assessment (CRA) framework^27^^,^^28^ to calculate the proportions of deaths for MM attributable to asbestos exposure. This methodology included the identification of risk-outcome pairs with convincing or probable evidence based on research studies, summarizing the relative risk of asbestos exposure through systematic reviews, and estimating exposure levels and distributions using spatiotemporal Gaussian process regression, DisMod-MR 2.1, and other relevant methods. Subsequently, the population attributable fractions (PAFs) and attributable burdens were calculated. Finally, PAFs and attributable burden for the combinations of risk factors were estimated taking into account the mediation of different risk factors through other risk factors.^18^

### 2.3 Joinpoint regression analysis

We used the joinpoint regression model to examine the changing temporal trends in MM incidence and mortality in China for the period 1990–2019. The joinpoint regression model was used to quantify time trends in a structured manner and test which trends between joinpoints are statistically significant.^29^ The overall trends in MM burden were reflected by the annual percentage change (APC), average APC (AAPC), and their respective 95% confidence intervals (CIs) between successive joinpoints in the age-standardized incidence (ASIR), age-standardized mortality (ASMR), and age-standardized DALYs (ASDR) rates. The Monte Carlo permutation method was used to test significant differences.^30^

### 2.4 Age-period-cohort analysis

Age-period-cohort analysis aims to figure out the independent effects of changes in the outcome risk associated with age groups, different periods, and birth cohorts. Therefore, an age-period-cohort analysis was conducted using a log-linear Poisson regression model based on successive 5-year age groups and consecutive 5-year periods from 1990 to 2019. Cases under 20 years of age were excluded from the age-period-cohort analysis due to their very small number. We also excluded cases aged over 95 years old cause they were recorded as one age group in the GBD 2019 database. The age effect was evaluated on longitudinal age-specific incidence and mortality rates in the reference cohort adjusted for period deviations. The period effect assessment was based on rate ratios (RRs) relative to the reference period and cohort effect was assessed by RRs relative to the reference cohort. The R package used in this study to conduct the model for age-period-cohort analysis was developed by the Biostatistics Branch of National Institutes of Health, USA.^31^

In the age-period-cohort analysis, approximate linearity was found in all effects, therefore we used a second order random walk (RW2) priors for all projection models (Supplementary Fig. 1).^32^^,^^33^

### 2.5 Bayesian age-period-cohort analysis

We predicted the numbers of new cases and deaths from MM between 2020 and 2029 by the Bayesian age-period-cohort (BAPC) analysis using integrated nested Laplace approximations (INLA) (R packages BAPC and INLA) because of its higher accuracy.^34^^,^^35^

In short, the BAPC model is considered a log-linear Poisson model:

(1) $$log\left(\lambda_{ij}\right)=\mu +\alpha_{i}+\beta_{j}+\gamma_{k}+z_{ij}.$$

In the model, *μ* represents the intercept, *α_i_, β_j_, γ_k_* represent the effects of age, period, and cohort, respectively, and *z_ij_* represents the parameters for additional unstructured heterogeneity. Priors for the age, period, and cohort effects were selected based on age-period-cohort analysis results; and overdispersion was adjusted by an RW2 or Gaussian prior, selected based on proximal model fit (modified BAPC models, BAPC-M) (Supplementary Table 1).

### 2.6 Model validation

Predictive performance of the BAPC model was assessed during a validation period of 2015–2019. We used different methods to forecast cases, deaths, and DALYs and compared their results to the observed values in the same period. The Nordpred package in R was used to conduct Nordpred APC analyses (Nordpred model). BAPC models using RW2 priors (BAPC-M1) and identically independently distributed (IID) prior (BAPC-M2) to account for overdispersion were conducted. Furthermore, we calculated the absolute number of events projected under three scenarios: stable rates (constant), a yearly decrease of 1% (optimistic), and a yearly increase of 1% (pessimistic), with reference to the observed rates in 2014. A lower root-mean-squared error (RMSE) indicates better predictive performance (Supplementary Table 2).

The BAPC-M model fitted best among age-period-cohort-based models with the observed incident, death, and DALYs counts during the validation period of 2015–2019. Optimistic method performed best in the prediction of female death; however, this deviation could be ascribed to a sudden change of trend during the chosen period (Supplementary Fig. 2). Therefore, we chose BAPC-M as our projection model.

The flowchart of our study is summarized in Supplementary Fig. 3. All statistical analyses were performed using the R (version 4.2.1) and a two-tailed *P* < 0.05 was considered statistically significant.

## 3 Results

### 3.1 Temporal trends of MM burden from 1990 to 2019

**Fig. 1:**
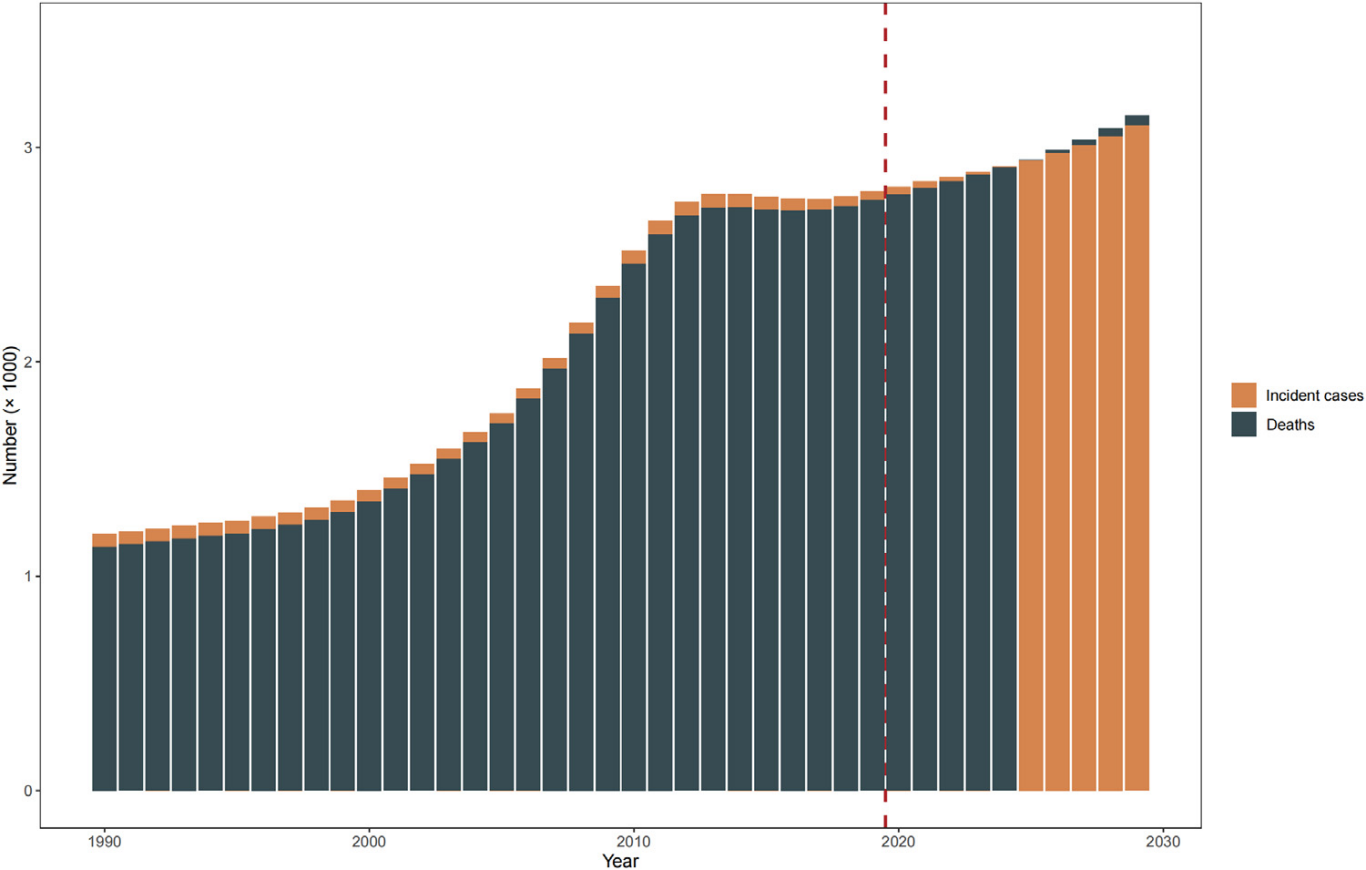
Estimated incident new cases and deaths during 1990–2019 and the projections to 2029.

**Fig. 2:**
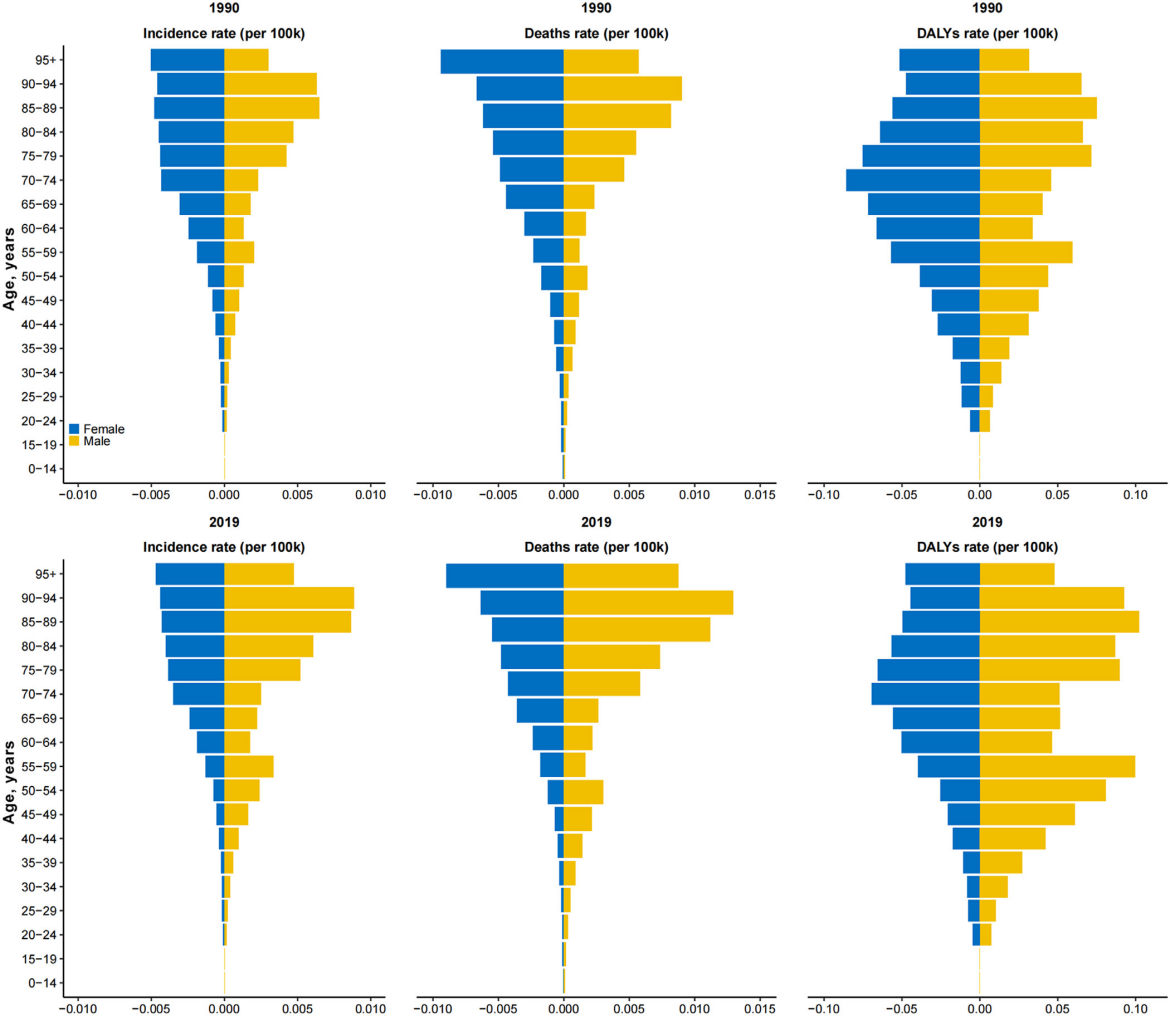
Rates of incidence, deaths and DALYs of malignant mesothelioma by age and sex in 1990 and 2019 in China. DALYs, disability-adjusted life-years.

The number of MM incident cases, deaths, and DALYs elevated significantly in the Chinese population from 1990 to 2019 (Fig. 1). Over the past three decades, the number of incident cases and deaths has more than doubled, increasing from 1193 to 2815 and from 1134 to 2773, respectively. Although the pace has slowed, this upward trend will be maintained through 2029. Regarding the proportion of incident cases, we found a roughly 6% increase for those who aged > 70 years (30% in 2019 versus 24% in 1990) but 10% decrease (21% in 2019 versus 31% in 1990) for those who aged < 50 years. As for the proportion of deaths, we found a similar increase for those who aged > 70 years but a decrease for those who aged < 50 years. However, we did not find a statistically significant change in ASIR (from 1.3 [95% UI, 1.0–1.9] in 1990 to 1.4 [95% UI, 1.2–1.7] in 2019), ASMR (from 1.3 [95% UI, 1.0–1.9] in 1990 to 1.4 [95% UI, 1.2–1.7] in 2019), and ASDR (from 36.8 [95% UI, 28.0–52.3] in 1990 to 39.4 [95% UI, 32.4–47.0] in 2019) from 1990 to 2019. Additionally, the ASIR, ASMR, and ASDR in men showed upward trends, increasing from 1.2 to 1.8 per million, 1.3 to 1.8 per million, and 34.9 to 50.9 per million, respectively. In contrast, the ASRs all showed downward trends in women (Fig. 2, Supplementary Tables 3–5).

**Fig. 3:**
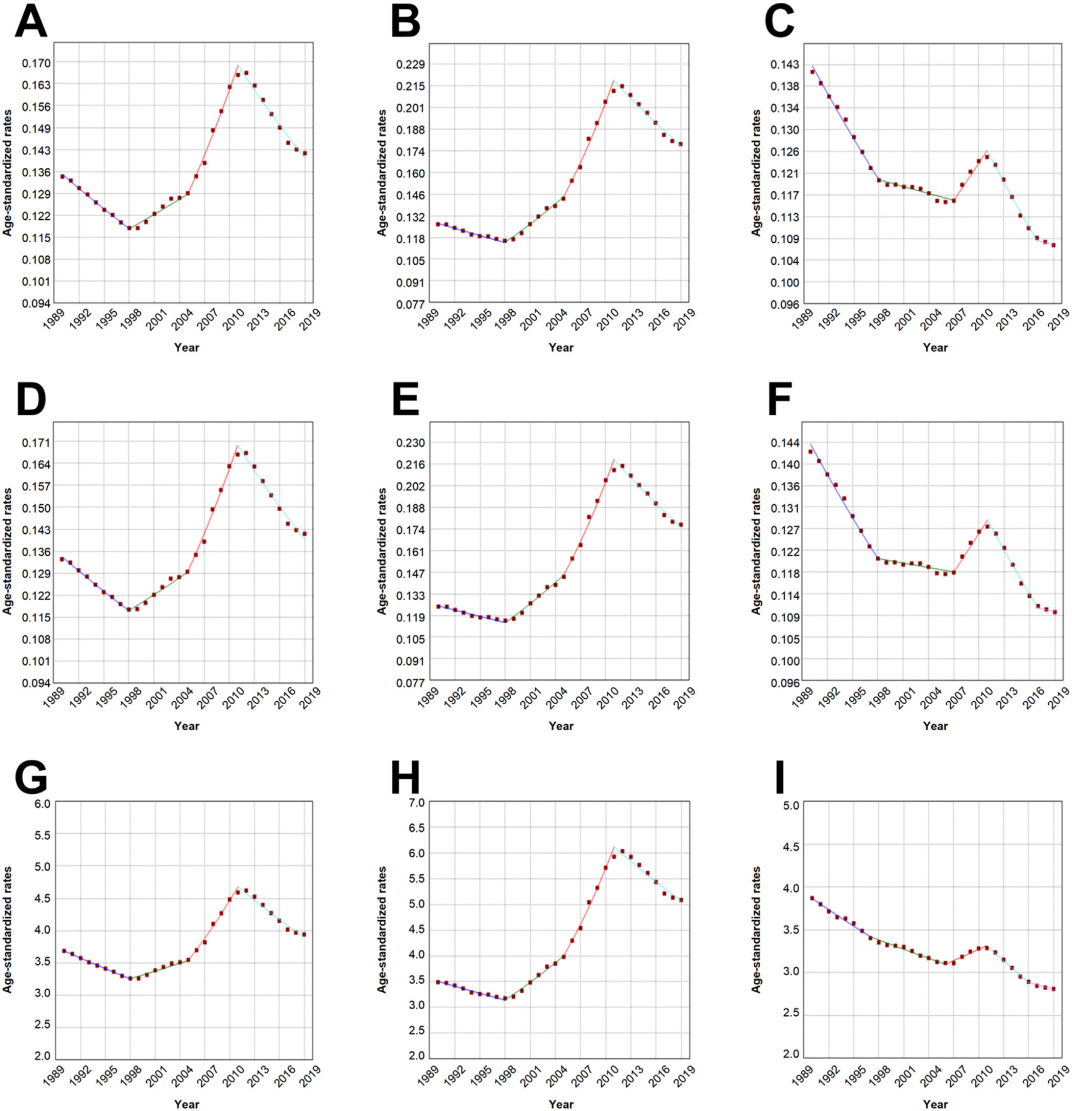
(A-I) Joinpoint analysis results of age-standardized incidence rates in both sexes (A), men (B), women (C); mortality rates in both sexes (D), men (E), women (F); disability-adjusted life-years rates in both sexes (G), men (H), women (I). *, indicates that the annual percent change is significantly different from zero at the alpha = 0.05 level.

Joinpoint regression analysis indicated that ASRs for MM marginally increased overall and for men (*P* *<* 0.05) during the observed period. However, we found a significant downward trend of ASRs in women (*P* < 0.05) (Fig. 3, Supplementary Table 6). The line of ASRs stayed stable until 2005, followed by a sharp increase and reached the peak in 2011 in all populations and both genders. Then all ASRs started to drop until 2019. In the past three decades, ASIR in women maintained a decreasing trend in most of the time, except for a temporary rise between 2007 and 2011 and a stagnation after 2017. The sharpest decrease of ASIR in women was present during 1990–1998 and 2011–2017. ASMR in women closely followed the ASIR, in both of which significant declining trends could be observed. ASDR in women is showing a moderate downward trend in recent years overall, with a fluctuation in the period of 2007–2011.

### 3.2 MM deaths attributable to occupational exposure to asbestos in China

We reviewed the proportions of deaths for MM attributable to occupational exposure to asbestos by sex and age from 1990 to 2019. It was observed that age-standardized mortality associated with asbestos exposure in both sexes first decreased and then started increasing around 2000s, following another decrease in the last 5 years. The proportion of men was lower than that of women at the beginning; however, the gap has been narrowing and finally surpassed women in 2009 (Supplementary Fig. 4). The effect of exposure factors on mortality of MM tended to increase with age regardless of gender (Supplementary Fig. 5).

### 3.3 Production and consumption of asbestos in China

**Fig. 4:**
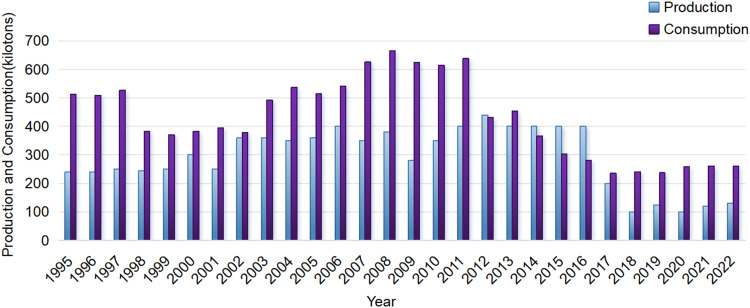
Asbestos production and consumption for China, 1995–2022.

Fig. 4 presents both production and consumption of asbestos in China during 1995–2022. Firstly, consumption is always higher than production. Secondly, production has steadily increased and reached a peak of 440 kilotons in 2012 but halved in 2017 (with only 200 kilotons). Thirdly, the consumption reached a peak of 660 kilotons in 2008, but dramatically dropped to 430 kilotons in 2012 and further dropped to only 230 kilotons in 2017. Afterwards the consumption remained stable with roughly 250 kilotons.

### 3.4 Projection of the burden of MM in China through 2029

**Fig. 5:**
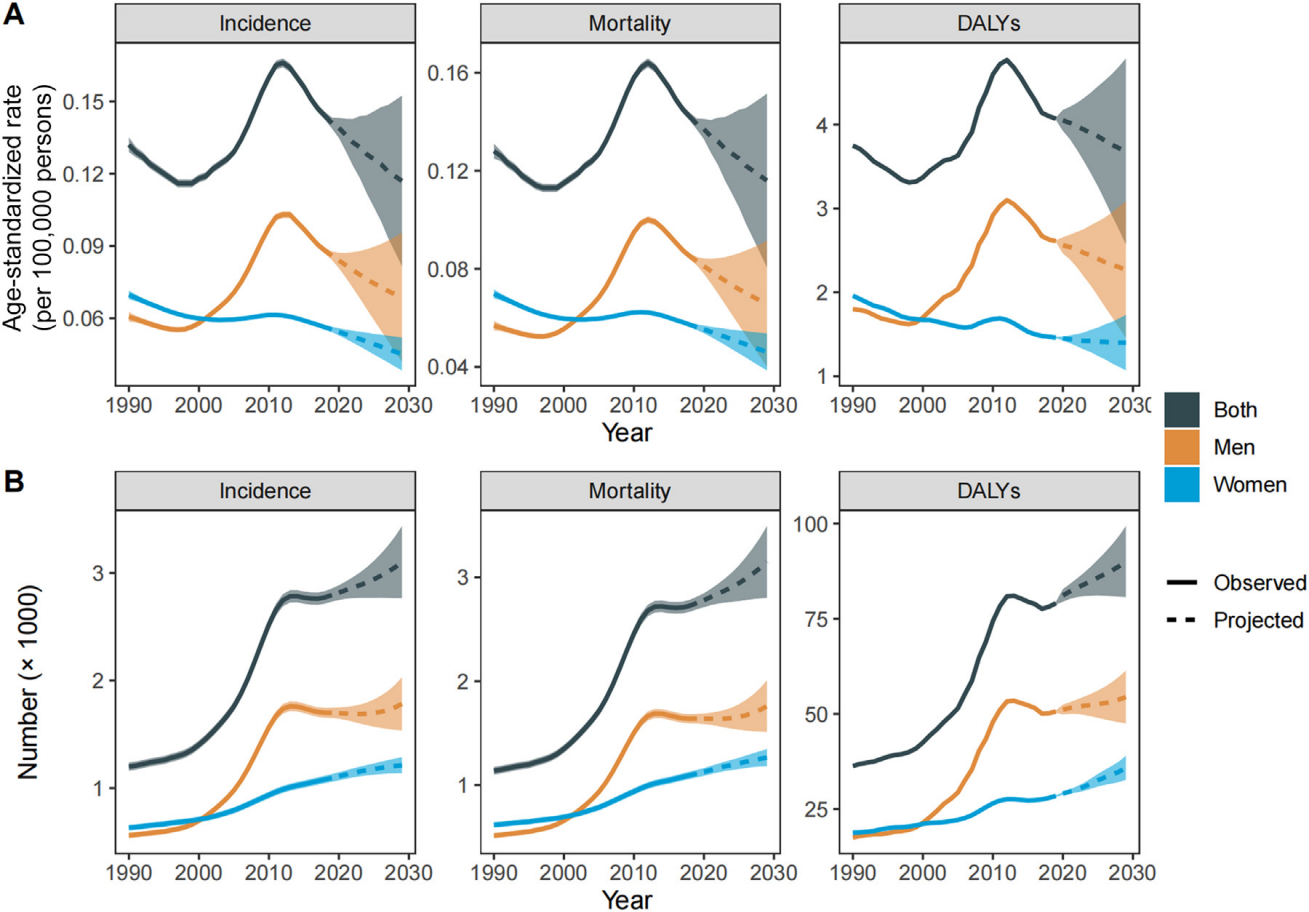
(A) The increasing trends of age-standardized rates of incidence, mortality and DALYs per 100 000 person-years of malignant mesothelioma in China, 1990–2029. (B) Counts of incidence, mortality, and DALYs due to malignant mesothelioma in China, 1990–2029. DALYs, disability-adjusted life-years.

Based on the GBD 2019 data from 1990 to 2019, we further predicted the ASRs and the estimated number of incident cases, deaths, and DALYs through 2029. In our previous validation, the ASRs calculated using BAPC-M models were nearly identical to the observations during 2015–2019 (Supplementary Fig. 6). In the next 10 years in China, it is predicted that the number of incident cases from mesothelioma will increase by 11% and reach 3101. The projections for deaths also will increase by 14% and reach 3149 and for DALYs will increase by 14%. We found a declining temporal trend in the next decade for age-standardized incidence rate, mortality rate and DALYs rate, with estimated annual percentage changes reaching −1.9% (95% CI, −1.9% to −1.8%), −1.8% (95% CI, −1.9% to −1.8%) and −1.0% (95% CI, −1.1% to −1.0%), respectively (Fig. 5, Table 1).

**Table 1:** Burden of MM in China projected until 2029.

Years	Sex	Incident cases (95% UI)	ASIR per 1,000,000 (95% UI)	EAPC of ASIR, 2029 vs. 2019 (95% UI)	Deaths (95% UI)	ASMR per 1,000,000 (95% UI)	EAPC of ASMR, 2029 vs. 2019 (95% UI)	DALYs (95% UI)	ASDR per 1,000,000 (95% UI)	EAPC of ASDR, 2029 vs. 2019 (95% UI)
2019	Both	2795 (2734, 2857)	1.4 (1.4, 1.4)	/	2753 (2693, 2814)	1.4 (1.4, 1.4)	/	79099 (78706, 79492)	40.7 (40.6, 40.8)	/
	Men	1700 (1652, 1748)	0.9 (0.8, 0.9)	/	1639 (1593, 1686)	0.8 (0.8, 0.8)	/	50621 (50307, 50935)	26.1 (26.0, 26.2)	/
	Women	1095 (1059, 1132)	0.6 (0.5, 0.6)	/	1111 (1075, 1148)	0.6 (0.6, 0.6)	/	28470 (28246, 28693)	14.6 (14.5, 14.7)	/
2029	Both	3101 (2766, 3436)	1.2 (0.8, 1.5)	−1.9 (−1.9, −1.8)	3149 (2804, 3494)	1.2 (0.8, 1.5)	−1.8 (−1.9, −1.8)	90043 (80708, 99379)	36.8 (25.7, 47.9)	−1.0 (−1.1, −1.0)
	Men	1784 (1536, 2031)	0.7 (0.4, 1.0)	−2.3 (−2.3, −2.2)	1761 (1512, 2010)	0.7 (0.4, 0.9)	−2.3 (−2.4, −2.3)	54447 (47472, 61422)	22.7 (14.5, 30.8)	−1.4 (−1.4, −1.3)
	Women	1213 (1138, 1288)	0.5 (0.4, 0.5)	−2.0 (−2.1, −2.0)	1265 (1183, 1347)	0.5 (0.4, 0.5)	−2.0 (−2.0, −1.9)	35853 (32688, 39017)	14.0 (10.7, 17.3)	−0.4 (−0.5, −0.3)

Abbreviations: ASDR, age-standardized DALYs rate; ASIR, age-standardized incidence rate; ASMR, age-standardized mortality rate; DALYs, disability-adjusted life years; EAPC, estimated annual percentage change; UI, uncertainty intervals.

## 4 Discussion

We found, for first time using GBD data on the Chinese population, that the burden of MM has been significantly increasing in China over the last three decades. We observed a significant elevation in incident new cases and deaths over the last 3 decades, increasing from 1193 in 1990 to 2815 in 2019 for incident cases and from 1134 in 1990 to 2773 in 2019 for death cases. We found a roughly 6% increase in the proportion of incident cases for those aged > 70 years (30% in 2019 versus 24% in 1990), while for the proportion of deaths similar elevation for those aged > 70 years was also found. Additionally, men had significantly higher DALYs due to MM across age groups compared to women. By 2029, the projected age-standardized rate for incidence and mortality are expected to reach 1.2 per million for both. While asbestos consumption is always higher than production, asbestos consumption reached a peak of 660 kilotons in 2008 and dramatically dropped to bottom with 230 kilotons in 2017 and afterwards the consumption remained stable with roughly 250 kilotons.

Our results suggested that around 3000 new cases and deaths arose each year in China in the past 30 years. However, real data might exceed the suggested figure. A study reviewed the Third National Cancer Survey (TNCS) and found that about 35% of the underlying cause of cancer death was inaccurate.^36^ MM did not have its own code in the ICD until 1999, before which MM was coded as malignant neoplasm of the pleura, malignant tumor of the trachea bronchus and lung, or malignant neoplasm without specification of site.^37^ However, prior studies found that even given its own code in ICD-10, the percentage of MM that is incorrectly classified remains at around 20–25%.^38^ Besides, in the past few decades, China has undergone enormous demographic and epidemiological transitions.^39^^,^^40^ Compared with health data in 1990, the quantity and quality of health data available in the most recent years have been optimized a lot, which may improve the accuracy of the estimated trends in burden of MM.^14^

The age-standardized rates of MM marginally increased from 1990 to 2019 but a significant decreasing trend arose from 2011. However, when separating sexes, the ASIR, ASMR, and ASDR on women all showed downward results. The recent declining trend is consistent with worldwide studies.^41^ However, considering that asbestos usage has not been completely banned in China, this decrease may be the result of enhanced protective measures and advancements in medical treatments leading to reduced mortality rates. Additionally, given that asbestos mining and consumption in China were approximately twice as high around 1980 compared to the following years, the high ASRs observed around 2011 could be a peak resulting from this historical exposure.^42^

The proportions of deaths for MM attributable to occupational exposure to asbestos in China remains high regardless of gender and elevated in the past 30 years among men. A worldwide study reported that the ASDR of MM attributable to occupational asbestos exposure was positively associated with socio-demographic index (SDI) at the national levels, with the proportion in most high-income regions reaching up to more than 90%.^43^ Since MM onset has a 20–40 years latency period after asbestos exposure,^44^ this result should be comprehended in conjunction with the historical combination of the last century. MM was very rare before the 1950s, and the link between asbestos and MM was first discovered in 1960.^45^ Several studies found that high mesothelioma mortality rates are regularly recorded in areas with a history of shipbuilding, asbestos cement industries, and oil refineries, suggesting that the MM burden increased as industrial manufacturing developed and consequent asbestos exposure.^38^^,^^46^^,^^47^

It is well known that asbestos is one of the most important occupational carcinogens and is classified as Group I carcinogen by International Agency for Research on Cancer (IARC). Nordic countries such as Sweden and Finland banned all types of asbestos use in the 1970s, leading to a declining trend of MM burden.^48^ However, chrysotile asbestos is still widely used in BRIC countries (Brazil, Russia, India, and China), which may lead to a continuing upward trend in age-adjusted mesothelioma incidence and mortality rates in the upcoming years.^7^ Therefore, regulatory policies targeting pure chrysotile and mixtures that include amphibole are necessary.^8^ It can be deduced that most deaths from MM should be preventable because most cases are associated with exposure to asbestos.^37^ There is growing evidence that reductions in asbestos consumption are causally associated with the declines in MM incidence,^6^^,^^49^ though the latency for declining will take at least 2 decades, according to the study from our group.^41^

We found, for the first time, that asbestos consumption in China dramatically dropped to 430 kilotons in 2012 and further dropped to only 230 kilotons in 2017, which is apparently attributed to China's major change of policy on asbestos use, i.e., China eliminated the use of amphibole asbestos and other types of asbestos except chrysotile asbestos in 2013^49^ and additionally in 2014 regulated the use of chrysotile asbestos and issued admission standard on chrysotile asbestos industry.^50^ We found asbestos consumption is always higher than production (Fig. 4), which suggests there is asbestos import for China domestic use. Based on the fact that the latency for the development of MM will take at least 2 decades, we could expect a higher incidence of MM in China as the production of asbestos reached its peak in year 2012, which is consistent with our findings that new cases of MM will be higher in the year 2029.

Compared to studies in other regions, the ASIR, ASMR, and ASDR of MM among men in China was notably low in 1990, with small sex differences observed.^51^, ^52^ Considering the very long latency of MM (20–50 years), it is possible that asbestos use was started in China around 1950s,^42^ which supports our finding of low male rates in 1990 and small sex difference. By 2019, the ASRs of MM in men greatly exceeded those in women and these circumstances will continue until 2029. As previously reported, a marked difference was also found among the two sexes in worldwide research, with the male population having a significantly higher incidence of MM.^53^, ^54^, ^55^ In 2019, men had 3-fold higher ASIR compared to women, and also had significantly higher ASMR and ASDR, compared to those in women.^43^ Besides, the global ASIR of MM declined in women but remained relatively stable in men from 1990 to 2019, which is similar to the trend from our results. This consequence may be a result of the predominance of male workers in jobs with high exposure to asbestos, such as shipbuilding and mining.

Mesothelioma incident cases and deaths vary widely across age groups, while age has also been identified in multiple studies as a predictive factor in survival.^56^ A recent study reported that the ASIR of MM in the United States increased from 0.5 to 1.24 cases per 100,000 people over age 60 years and reached 6.34 cases per 100,000 people in the age group older than 85 years.^7^ Besides, all new cases identified in Western Europe from 1990 to 2017 were among individuals older than 70 years.^41^ The divergences found between the age groups are possibly due to the long latency period of MM, resulting in the lower burden among younger cohorts. Moreover, younger patients with MM occurred more frequently in women and had longer survival as compared with older patients, suggesting that MM may be biologically different between younger and older patients.^57^^,^^58^ Young patients are unlikely to suffer from MM due to occupational exposure to asbestos. Instead, genetic predisposition and environmental exposure may be the etiology.^59^ Nowadays in China, the medical system has made enormous strides, and the population is getting larger and older. Thus, despite the decrease in ASIR, ASMR, and ASDR in the next decade, the overall number of new cases and deaths caused by MM in China per year will continue to increase slightly.

Our study has several limitations. Firstly, although we have used various mathematical models to correct the data, there may still be incompatibilities because the data provided by GBD 2019 come from different locations, which may introduce bias due to uneven development between regions, leading to a potential underestimation of the burden of the disease. Secondly, the GBD database did not provide detailed information on the genetic factors and sub-types of MM (pleural mesothelioma and peritoneal mesothelioma). Thirdly, we did not consider the impact of the COVID-19 pandemic on the burden of MM. However, cancer patients are more susceptible to infection than the general population^60^^,^^61^ and the subsequent economic and health impact of COVID-19 cannot be ignored.

Future research could investigate the sources of asbestos exposure causing MM in China. Previous studies have shown significant differences between male and female patients with regard to different types of asbestos exposure (primary exposure: asbestos workers; and secondary exposure: domestic, environmental, or both).^62^ Most males experience primary asbestos exposure, while females exhibit significantly higher rates of secondary asbestos exposure. The higher proportion observed in female patients may indicate the significance of environmental exposure. Therefore, future research could focus on distinguishing the types of exposure and place more emphasis on the burden of MM caused by the risks of indirect asbestos exposure. Additionally, MM is often misdiagnosed. By incorporating advanced data analysis techniques, future studies may provide novel methods to estimate misdiagnosis rates, correct calculations, and produce optimized results.

## 5 Conclusions

In summary, we found, for the first time using GBD data on the Chinese population, that the burden of MM has been significantly increasing in China over the last three decades and will continue to increase in the upcoming decade. We found asbestos consumption is always higher than production, which suggests there is asbestos import for China domestic use. Asbestos consumption reached a peak of 660 kilotons in 2008 and dramatically dropped since 2012 and reached bottom in 2017 with 230 kilotons, which is attributed to China's major change of policy on asbestos use in 2013–2014. Additionally, we also found the proportion of age-standardized mortality attributable to occupational exposure to asbestos remains high, suggesting an urgent need for a complete ban on chrysotile asbestos in China.

## Declaration of competing interest

The authors declare that they have no known competing financial interests or personal relationships that could have appeared to influence the work reported in this paper.

## Appendix Supplementary materials

Image, application 1
