# Supplementary material for: Burden of malignant mesothelioma in China during 1990–2019 and the projections through 2029
Source: J Natl Cancer Cent. 2024 May 11;4(3):214–22. doi: 10.1016/j.jncc.2024.05.003 (PMC11401487; doi:10.1016/j.jncc.2024.05.003)
Supplement: Supplementary file 1 [file mmc1.pdf]

Supplementary Materials

Burden of malignant mesothelioma in China during 1990-2019 and the projections through 2029

Qiulin Huang, Youli Chen, Liyou Lian, Qiqi Lei, Jinfei Chen, Licun Wu, Kari Hemminki, Jianguang Ji, Tianhui Chen

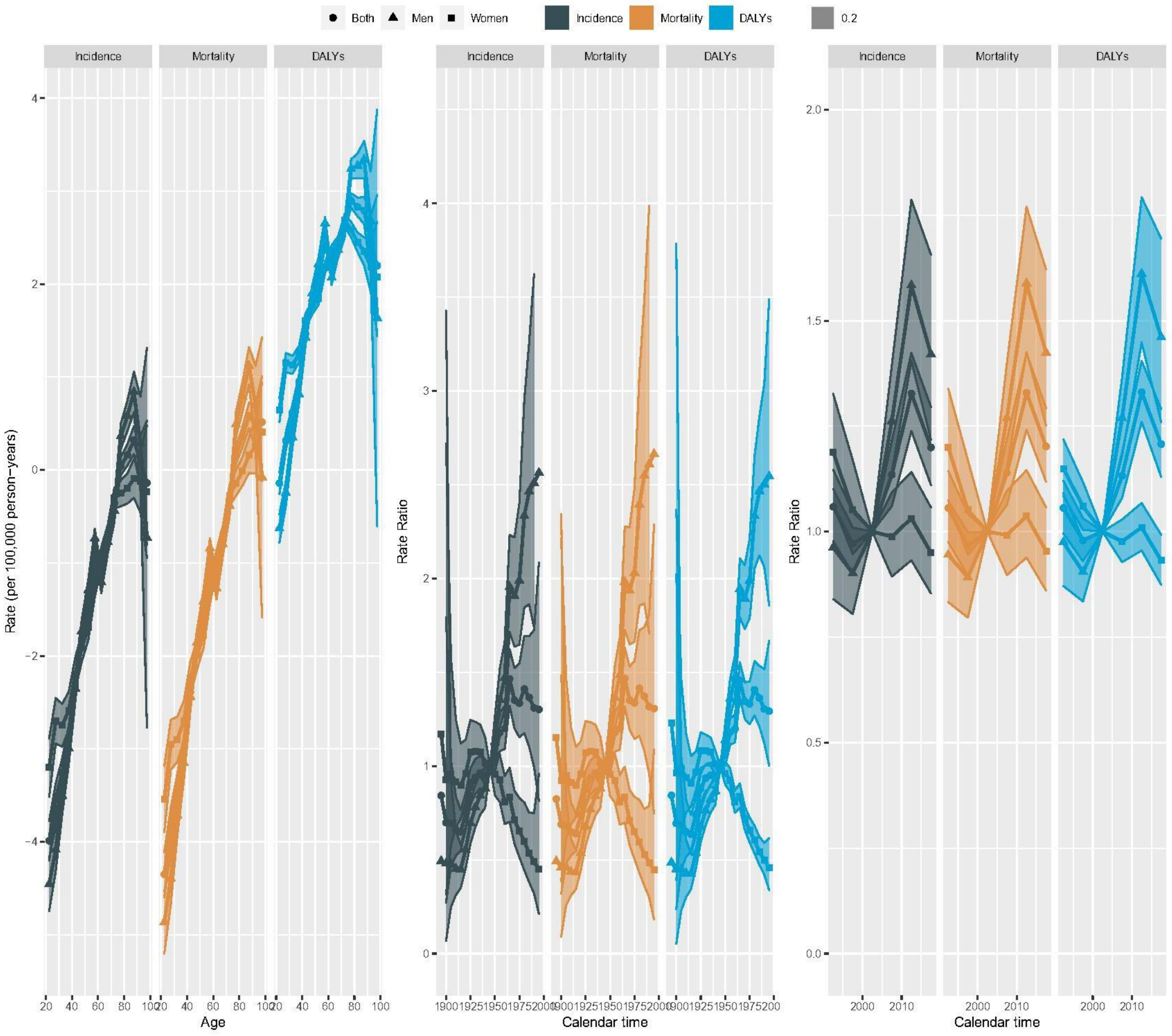

Supplementary Fig.1. Age-Period-Cohort analysis results. Left panel, longitudinal age; middle panel, cohort effect; right panel, period effect.

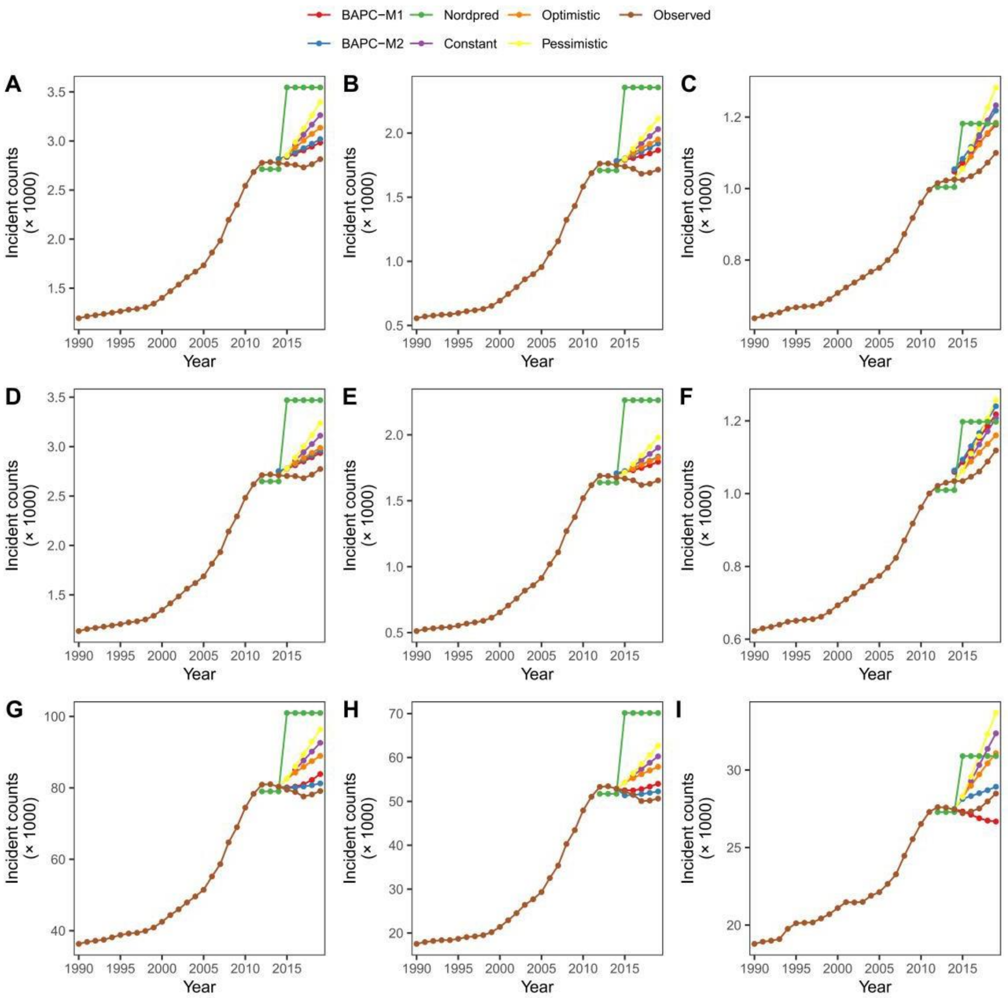

Supplementary Fig. 2. Malignant mesothelioma burden projection model validation. (A-C) Incident cases in both sexes (A), men (B), women (C). (D-F) Deaths in both sexes (D), men (E), women (F). (G-I) Disability-adjusted life-years in both sexes (G), men (H), women (I). BAPC-M1, a second order random walk prior for overdispersion; BAPC-M2, identically independently distributed prior for overdispersion; Nordpred model, Nordpred age-period-cohort analyses run by Nordpred package in R; Constant, rates remained stable with reference to the 2014 observed rates; Optimistic, decreased annually by 1% from 2014; Pessimistic, increased annually by 1% from 2014.

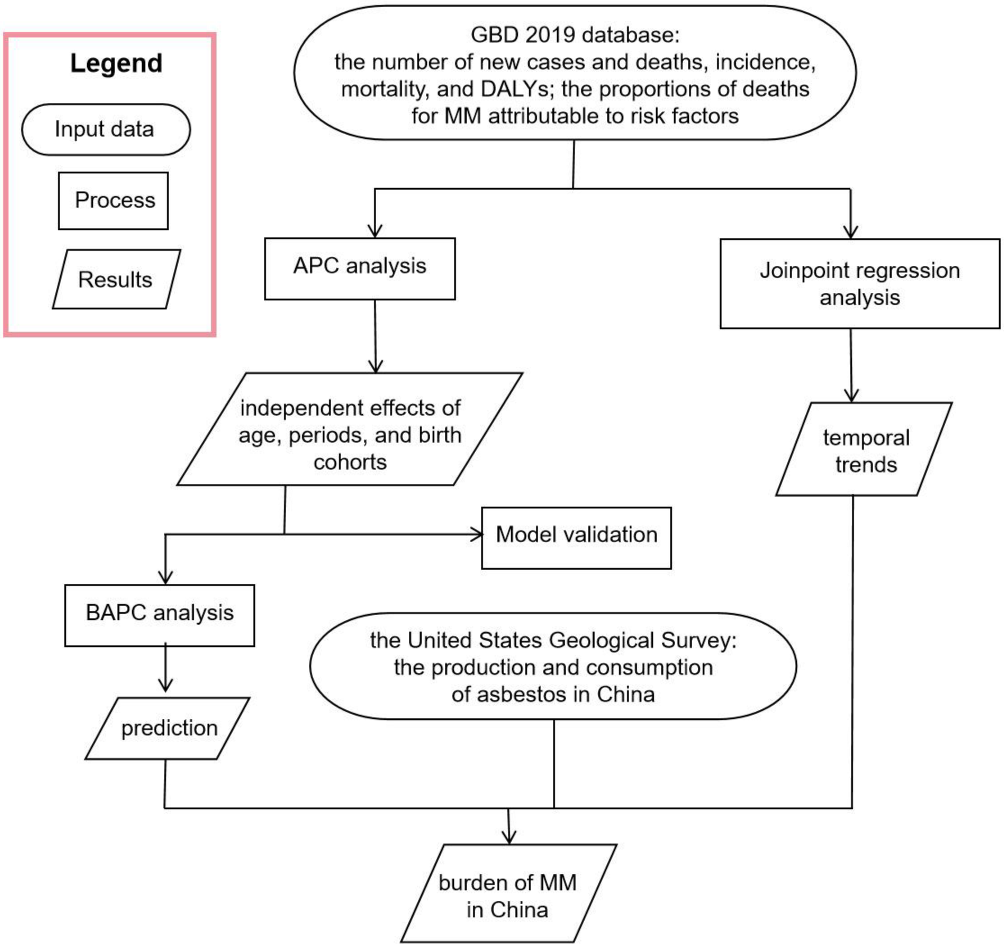

Supplementary Fig. 3. Study flowchart. APC, age-period-cohort; BAPC, age-period-cohort; DALYs, disability-adjusted life-years; GBD, Global Burden of Disease; MM, malignant mesothelioma.

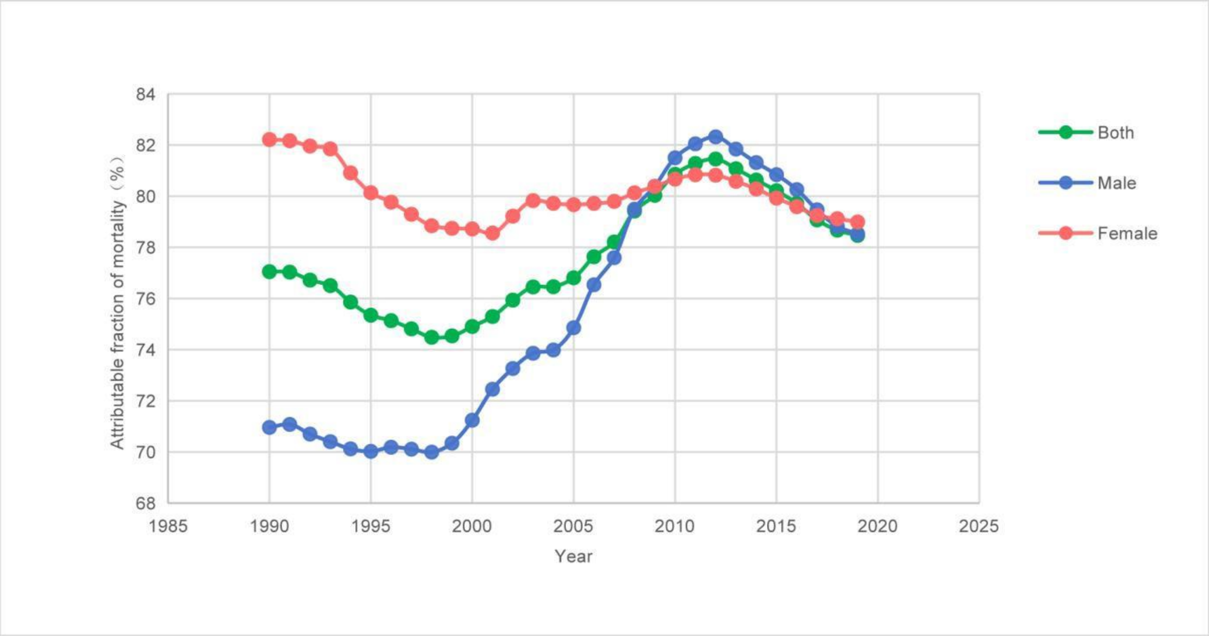

Supplementary Fig. 4. The proportions of age-standardized mortality for malignant mesothelioma attributable to occupational exposure to asbestos in China, 1990-2019.

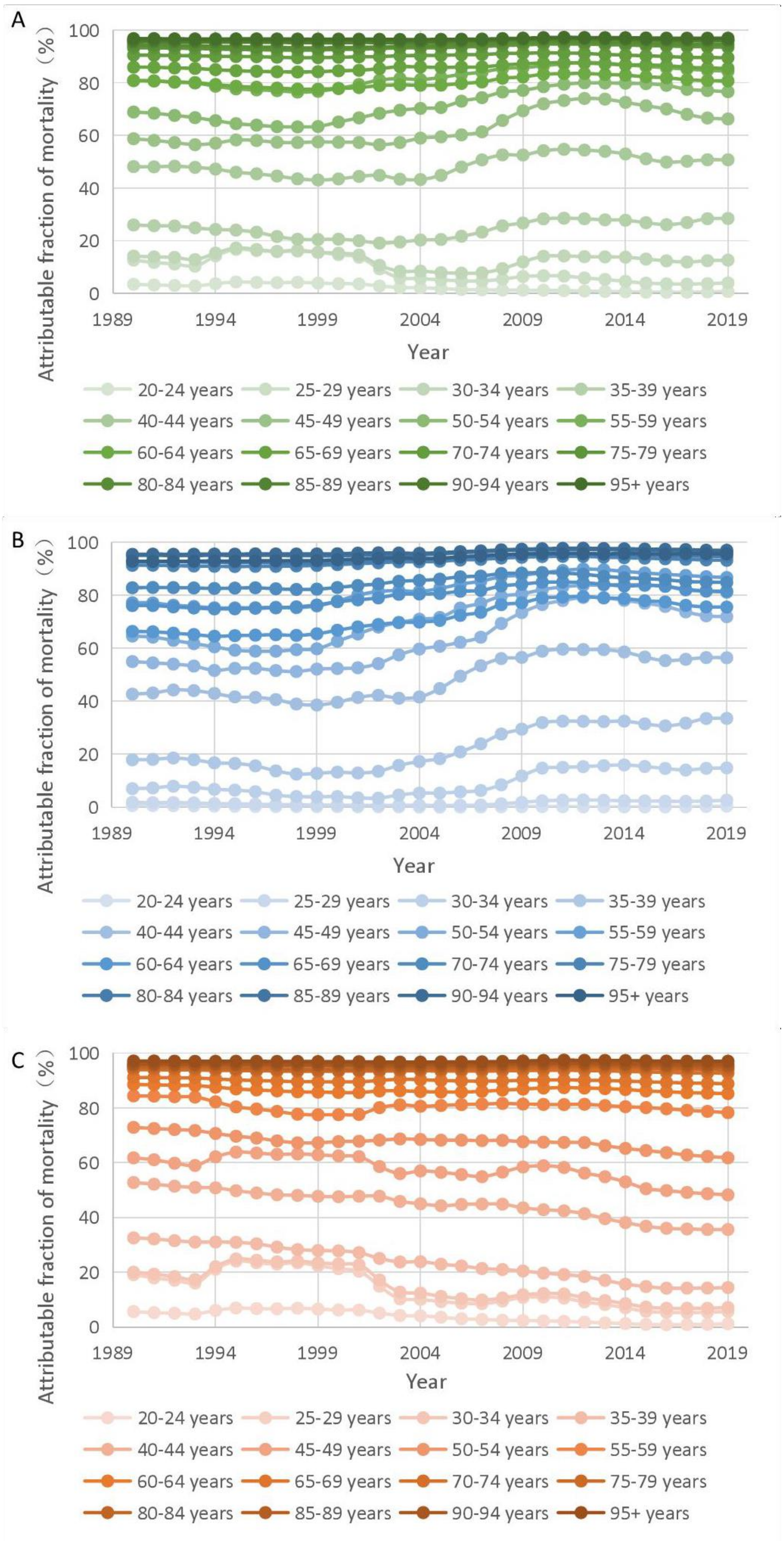

Supplementary Fig. 5. (A-C)The proportions of deaths attributable to occupational exposure to asbestos by age in China, 1990-2019. Proportions in both sexes (A), men (B), women (C).

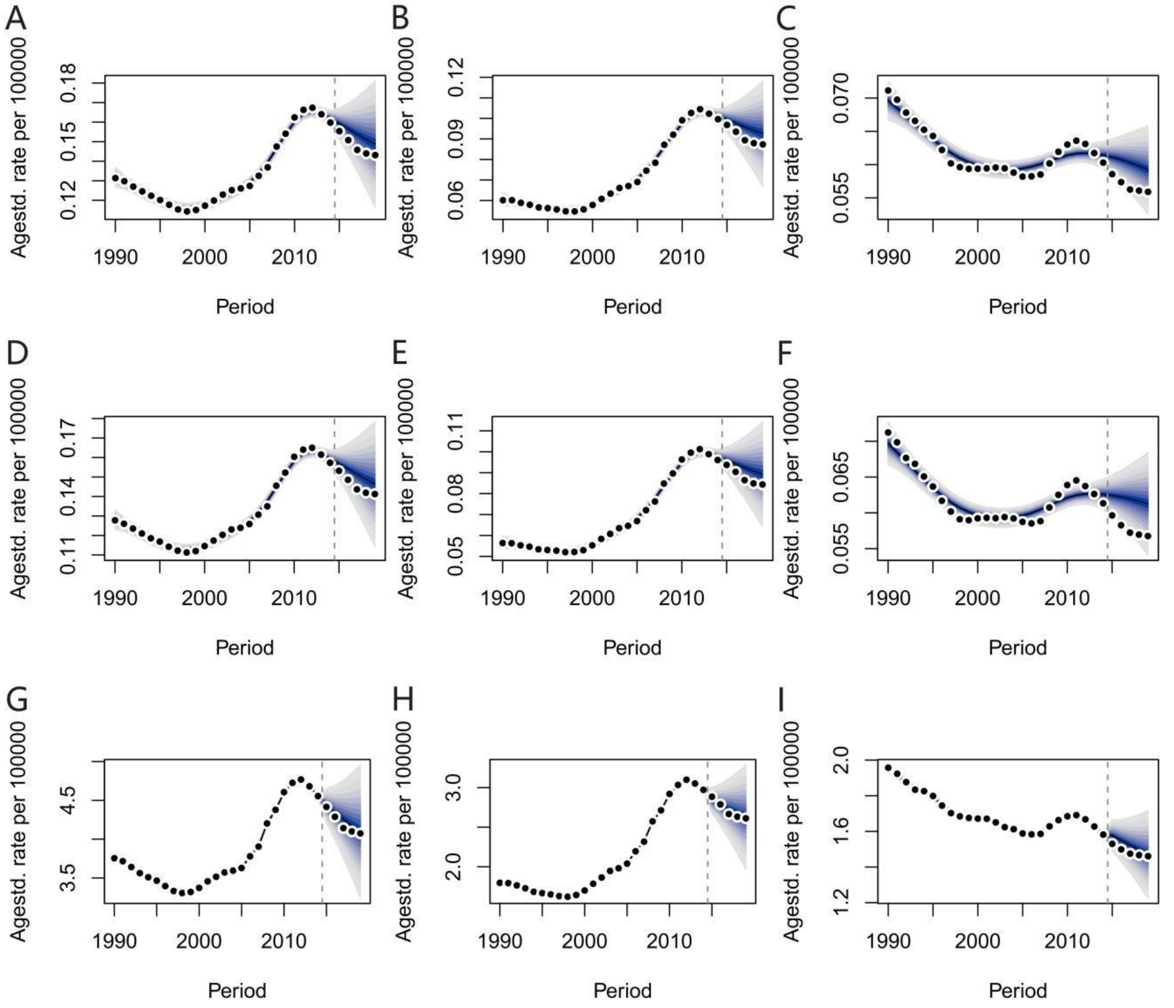

Supplementary Fig. 6. Malignant mesothelioma burden retrospective projection during the validation period of 2015-2019. (A-C) Incident cases in both sexes (A), men (B), women (C). (D-F) Deaths in both sexes (D), men (E), women (F). (G-I) Disability-adjusted life-years in both sexes (G), men (H), women (I).

Supplementary Table 1

Model parameters for the modified Bayesian Age-Period-Cohort analysis (BAPC-M).

Data type	Population	Age	Period	Cohort	Overdispersion
Incidence	Both sexes	RW2	RW2	RW2	RW2
	Men	RW2	RW2	RW2	RW2
	Women	RW2	RW2	RW2	RW2
Mortality	Both sexes	RW2	RW2	RW2	RW2
	Men	RW2	RW2	RW2	RW2
	Women	RW2	RW2	RW2	RW2
DALYs	Both sexes	RW2	RW2	RW2	IID
	Men	RW2	RW2	RW2	IID
	Women	RW2	RW2	RW2	IID

Abbreviations: DALYs, disability-adjusted life-years; IID, identically independently distributed; RW2, a random walk of second order.

Supplementary Table 2

Root-mean-squared errors for comparison of malignant mesothelioma projection models.

Data types	Population	Projection models					
		BAPC-M1	BAPC-M2	Constant	Optimistic	Nordpred	Pessimistic
Incident cases	Both	0.147	0.172	0.324	0.249	0.780	0.401
	Men	0.122	0.157	0.227	0.181	0.646	0.276
	Women	0.071	0.097	0.097	0.069	0.129	0.126
Deaths sexes	Both	0.144	0.160	0.248	0.178	0.755	0.322
	Men	0.114	0.141	0.180	0.136	0.618	0.225
	Women	0.085	0.100	0.069	0.043	0.131	0.097
DALYs sexes	Both	3.266	2.040	9.759	7.641	22.341	11.957
	Men	2.444	1.352	6.957	5.572	19.217	8.392
	Women	1.023	0.840	2.805	2.076	3.229	3.568

Abbreviations: BAPC-M1, a second order random walk prior for overdispersion; BAPC-M2, identically independently distributed prior for overdispersion; Nordpred model, Nordpred age-period-cohort analyses run by Nordpred package in R; Constant, rates remained stable with reference to the 2014 observed rates; DALYs, disability-adjusted life-years; Optimistic, decreased annually by 1% from 2014; Pessimistic, increased annually by 1% from 2014.

Supplementary Table 3

The number of incident cases and the incidence rates of malignant mesothelioma in China in 1990 and 2019 and the estimated annual percentage changes from 1990 to 2019.

Characteristics	1990		2019		1990-2019
	Incident cases No. ×10^3 (95% UI)	ASIR per 1,000,000 No. (95% UI)	Incident cases No. ×10^3 (95% UI)	ASIR per 1,000,000 No. (95% UI)	EAPC in ASIR No. (95% CI)
Overall	1193 (909, 1674)	1.3 (1.0, 1.9)	2815 (2313, 3346)	1.4 (1.2, 1.7)	0.9 (0.6, 1.3)
Sex					
Male	556 (432, 799)	1.2 (1.0, 1.8)	1715 (1319, 2158)	1.8 (1.4, 2.3)	2.3 (1.8, 2.8)
Female	637 (415, 1044)	1.4 (1.0, 2.3)	1100 (699, 1411)	1.1 (0.7, 1.4)	-0.7 (-0.8, -0.5)
Age [a]					
0 to 19	0 (0, 0)	0.0 (0.0, 0.0)	0 (0, 0)	0 (0, 0)	
20 to 24	35 (23, 55)	0.3 (0.2, 0.4)	20 (16, 25)	0.2 (0.2, 0.3)	
25 to 29	45 (27, 75)	0.4 (0.2, 0.7)	40 (32, 49)	0.4 (0.3, 0.4)	
30 to 34	46 (30, 71)	0.5 (0.3, 0.8)	68 (55, 81)	0.5 (0.4, 0.6)	
35 to 39	70 (48, 104)	0.8 (0.5, 1.1)	81 (63, 99)	0.8 (0.6, 1.0)	
40 to 44	86 (61, 122)	1.3 (0.9, 1.8)	133 (105, 164)	1.3 (1.0, 1.6)	
45 to 49	91 (67, 132)	1.8 (1.3, 2.5)	252 (196, 315)	2.1 (1.6, 2.6)	
50 to 54	112 (83, 169)	2.4 (1.7, 3.5)	379 (296, 474)	3.0 (2.4, 3.8)	
55 to 59	163 (122, 235)	3.7 (2.8, 5.4)	426 (332, 525)	4.5 (3.5, 5.5)	
60 to 64	127 (93, 181)	3.6 (2.6, 5.1)	272 (216, 327)	3.5 (2.7, 4.2)	
65 to 69	128 (94, 184)	4.7 (3.4, 6.7)	311 (250, 376)	4.4 (3.5, 5.3)	
70 to 74	122 (91, 173)	6.5 (4.8, 9.2)	277 (221, 337)	5.8 (4.6, 7.0)	
75 to 79	95 (74, 133)	8.3 (6.5, 11.6)	256 (208, 310)	8.6 (7.0, 10.4)	
80 to 84	49 (39, 70)	8.8 (6.8, 12.4)	180 (146, 216)	9.4 (7.6, 11.3)	
85 to 89	20 (15, 28)	10.2 (7.7, 14.4)	95 (77, 115)	11.2 (9.1, 13.5)	
90 to 94	4 (3, 5)	9.4 (7.1, 13.5)	22 (17, 28)	9.9 (7.4, 12.3)	
95+	1 (0, 1)	9.1 (5.9, 16.7)	4 (3, 5)	9.0 (6.3, 11.5)	

[a] Crude incidence rate in each age group.

Abbreviations: ASIR, age-standardized incidence rate; CI, confidence interval; EAPC, estimated annual percentage change; UI: uncertainty interval.

Supplementary Table 4

The number of deaths and the mortality rates of malignant mesothelioma in China in 1990 and 2019 and the estimated annual percentage changes from 1990 to 2019.

Characteristics	1990		2019		1990-2019
	Deaths cases No.×10^3 (95% UI)	ASMR per 1,000,000 No. (95% UI)	Deaths cases No.×10^3 (95% UI)	ASMR per 1,000,000 No. (95% UI)	EAPC in ASMR No. (95% CI)
Overall	1134 (865, 1589)	1.3 (1.0, 1.9)	2773 (2283, 3321)	1.4 (1.2, 1.7)	0.9 (0.6, 1.3)
Sex					
Male	512 (396, 742)	1.3 (1.0, 1.8)	1654 (1288, 2139)	1.8 (1.4, 2.2)	2.3 (1.8, 2.8)
Female	622 (413, 1015)	1.4 (0.9, 2.3)	1119 (724, 1444)	1.1 (0.7, 1.4)	-0.6 (-0.8, -0.4)
Age [a]					
0 to 19	0 (0, 0)	0.0 (0.0, 0.0)	0 (0, 0)	0.0 (0.0, 0.0)	
20 to 24	24 (16, 38)	0.2 (0.1, 0.3)	14 (11, 17)	0.2 (0.1, 0.2)	
25 to 29	34 (20, 56)	0.3 (0.2, 0.5)	30 (24, 37)	0.3 (0.2, 0.3)	
30 to 34	39 (26, 59)	0.4 (0.3, 0.7)	57 (46, 68)	0.4 (0.4, 0.5)	
35 to 39	62 (42, 93)	0.7 (0.5, 1.0)	71 (56, 87)	0.7 (0.6, 0.9)	
40 to 44	80 (58, 113)	1.2 (0.9, 1.7)	125 (99, 154)	1.2 (1.0, 1.5)	
45 to 49	81 (60, 116)	1.6 (1.2, 2.2)	227 (179, 288)	1.9 (1.5, 2.4)	
50 to 54	101 (73, 148)	2.1 (1.5, 3.1)	341 (265, 430)	2.7 (2.1, 3.4)	
55 to 59	148 (110, 212)	3.4 (2.5, 4.9)	388 (308, 482)	4.1 (3.3, 5.1)	
60 to 64	120 (88, 173)	3.4 (2.5, 4.9)	259 (205, 313)	3.3 (2.6, 4.0)	
65 to 69	125 (92, 181)	4.6 (3.4, 6.6)	307 (247, 376)	4.4 (3.5, 5.3)	
70 to 74	125 (93, 179)	6.7 (4.9, 9.5)	288 (229, 352)	6.0 (4.8, 7.4)	
75 to 79	105 (80, 149)	9.2 (7.0, 13.1)	287 (235, 347)	9.6 (7.9, 11.6)	
80 to 84	59 (46, 83)	10.5 (8.1, 14.7)	217 (179, 267)	11.4 (9.4, 14.0)	
85 to 89	25 (19, 35)	13.1 (9.9, 18.5)	123 (100, 149)	14.4 (11.7, 17.5)	
90 to 94	5 (4, 7)	13.7 (10.3, 19.9)	32 (24, 40)	14.4 (10.9, 18.0)	
95+	1 (0, 2)	17.1 (10.8, 31.6)	8 (5, 10)	17.2 (12.0, 21.9)	

[a] Crude mortality rate in each age group.

Abbreviations: ASMR, age-standardized mortality rate; CI, confidence interval; EAPC, estimated annual percentage change; UI, uncertainty interval.

Supplementary Table 5

The number of DALYs and the DALYs rates of malignant mesothelioma in China in 1990 and 2019 and the estimated annual percentage changes from 1990 to 2019.

Characteristics	1990		2019		1990-2019
	DALYs No. ×10^3 (95% UI)	Age-standardized DALYs rate per 1,000,000 No. (95% UI)	DALYs No. ×10^3 (95% UI)	Age-standardized DALYs rate per 1,000,000 No. (95% UI)	EAPC in Age-standardized DALYs rates No. (95% CI)
Overall	36320 (27230, 51581)	36.8 (28.0, 52.3)	79117 (65068, 94570)	39.4 (32.4, 47.0)	1.0 (0.6, 1.3)
Sex					
Male	17531 (13461, 25134)	34.9 (27.1, 50.5)	50636 (39117, 65807)	50.9 (39.4, 65.5)	2.4 (1.9, 2.9)
Female	18789 (11727, 30963)	38.7 (24.9, 63.4)	28481 (18307, 37081)	28.1 (18.1, 36.6)	-0.9 (-1.0, -0.8)
Age [a]					
0 to 19	0 (0, 0)	0.0 (0.0, 0.0)	0 (0, 0)	0.0 (0.0, 0.0)	
20 to 24	1620 (1058, 2514)	12.2 (8.0, 19.0)	945 (760, 1154)	11.5 (9.3, 14.1)	
25 to 29	2112 (1263, 3467)	19.2 (11.5, 31.5)	1884 (1478, 2311)	17.0 (13.4, 20.9)	
30 to 34	2210 (1468, 3334)	25.0 (16.6, 37.7)	3237 (2620, 3878)	25.1 (20.3, 30.0)	
35 to 39	3203 (2200, 4854)	35.0 (24.0, 53.0)	3672 (2922, 4511)	36.4 (29.0, 44.7)	
40 to 44	3773 (2709, 5316)	56.1 (40.3, 79.1)	5864 (4687, 7248)	57.7 (46.1, 71.3)	
45 to 49	3425 (2531, 4886)	66.2 (48.9, 94.5)	9571 (7549, 12150)	78.9 (62.2, 100.1)	
50 to 54	3781 (2753, 5535)	79.1 (57.6, 115.8)	12782 (9963, 16058)	102.2 (79.6, 128.4)	
55 to 59	4858 (3604, 6940)	111.8 (82.9, 159.7)	12716 (10092, 15787)	134.1 (106.4, 166.5)	
60 to 64	3371 (2478, 4892)	95.2 (70.0, 138.1)	7286 (5806, 8814)	92.8 (73.9, 112.2)	
65 to 69	2952 (2175, 4290)	107.9 (79.5, 156.8)	7242 (5826, 8854)	102.9 (82.8, 125.8)	
70 to 74	2432 (1802, 3479)	129.0 (95.5, 184.5)	5565 (4425, 6809)	116.3 (92.5, 142.3)	
75 to 79	1613 (1239, 2289)	141.3 (108.6, 200.6)	4411 (3612, 5314)	147.8 (121.0, 178.0)	
80 to 84	702 (544, 988)	124.6 (96.5, 175.2)	2562 (2112, 3147)	134.4 (110.8, 165.1)	
85 to 89	229 (173, 325)	119.2 (90.1, 169.6)	1111 (908, 1350)	130.6 (106.8, 158.7)	
90 to 94	36 (27, 53)	97.4 (73.1, 141.9)	228 (172, 284)	101.6 (76.5, 126.5)	
95+	6 (4, 11)	93.7 (59.2, 173.0)	41 (28, 52)	91.8 (63.8, 117.5)	

[a] Crude DALYs rate in each age group.

Abbreviations: ASMR, age-standardized mortality rate; CI, confidence interval; DALYs, disability-adjusted life-years; EAPC, estimated annual percentage change; UI, uncertainty interval.

Supplementary Table 6

Joinpoint analysis results.

	Both sexes			Men			Women		
	Period	APC/AAPC (95% CI)	*P*-values	Period	APC/AAPC (95% CI)	*P*-values	Period	APC/AAPC (95% CI)	*P*-values
Incidence	1990~1998	-1.7 (-2.1, -1.3)	< 0.001	1990~1998	-1.3 (-1.8, -[illegible]	< 0.001	1990~1998	-2.1 (-2.3, -1.9)	< 0.001
	1998~2005	1.3 (0.8, 1.8)	< 0.001	1998~2005	3.3 (2.7, 3.9)	< 0.001	1998~2007	-0.4 (-0.5, -0.2)	< 0.001
	2005~2011	4.7 (4.3, 5.1)	< 0.001	2005~2011	7.2 (6.7, 7.7)	< 0.001	2007~2011	2.0 (1.6, 2.5)	< 0.001
	2011~2019	-2.4 (-2.6, -2.1)	< 0.001	2011~2019	-2.6 (-2.9, -[illegible]	< 0.001	2011~2017	-2.5 (-2.7, -2.3)	< 0.001
							2017~2019	-0.4 (-1.6, 0.8)	0.496
	Full Range	0.1 (0.0, 0.3)	0.155		1.1 (0.9, 1.4)	< 0.001		-1.0 (-1.1, -0.8)	< 0.001
Mortality	1990~1998	-1.7 (-2.1, -1.3)	< 0.001	1990~1998	-1.1 (-1.7, -[illegible]	< 0.001	1990~1998	-2.2 (-2.4, -2.0)	< 0.001
	1998~2005	1.4 (0.9, 1.9)	< 0.001	1998~2005	3.5 (2.9, 4.1)	< 0.001	1998~2007	-0.3 (-0.4, -0.1)	0.001
	2005~2011	4.6 (4.2, 5.0)	< 0.001	2005~2011	7.1 (6.6, 7.7)	< 0.001	2007~2011	2.2 (1.7, 2.6)	< 0.001
	2011~2019	-2.4 (-2.6, -2.2)	< 0.001	2011~2019	-2.7 (-3.1, -[illegible]	< 0.001	2011~2017	-2.5 (-2.7, -2.2)	< 0.001
							2017~2019	-0.3 (-1.5, 0.9)	0.56
	Full Range	0.1 (0.0, 0.3)	0.123		1.2 (0.9, 1.4)	< 0.001		-0.9 (-1.0, -0.8)	< 0.001
DALYs	1990~1998	-1.5 (-2.0, -1.1)	< 0.001	1990~1998	-1.4 (-1.9, -[illegible]	< 0.001	1990~1997	-1.7 (-2.0, -1.5)	< 0.001
	1998~2005	1.2 (0.7, 1.7)	< 0.001	1998~2005	3.5 (2.9, 4.1)	< 0.001	1997~2006	-1.1 (-1.2, -0.9)	< 0.001
	2005~2011	4.8 (4.4, 5.2)	< 0.001	2005~2011	7.3 (6.8, 7.8)	< 0.001	2006~2011	1.4 (1.1, 1.7)	< 0.001
	2011~2019	-2.3 (-2.5, -2.0)	< 0.001	2011~2019	-2.3 (-2.7, -[illegible]	< 0.001	2011~2016	-2.7 (-3.1, -2.4)	< 0.001
							2016~2019	-1.0 (-1.6, -0.3)	0.006
	Full Range	0.2 (0.0, 0.4)	0.037		1.3 (1.1, 1.5)	< 0.001		-1.1 (-1.2, -1.0)	< 0.001

Abbreviations: APC, annual percentage change; AAPC, average APC; CI, confidence interval; DALYs, disability-adjusted life-years.
